# Iron and energy metabolic interactions in Treg-mediated immune regulation

**DOI:** 10.3389/fimmu.2025.1554028

**Published:** 2025-03-19

**Authors:** Frédérique Savagner, Thomas Farge, Zoubida Karim, Meryem Aloulou

**Affiliations:** ^1^ Biochemistry Laboratory, University of Toulouse, Toulouse, France; ^2^ Inserm U1297, Institute of Metabolic and Cardiovascular Diseases (I2MC), Toulouse, France; ^3^ Biochemistry Laboratory, Genetic and Hormonal Department, Federative Institute of Biology, Academic Hospital, Toulouse, France; ^4^ INFINITy, Toulouse Institute for Infectious and Inflammatory Diseases, INSERM U1291, CNRS U5051, University of Toulouse, Toulouse, France

**Keywords:** Foxp3, Treg, mitochondria, iron, ROS, immunoregulation

## Abstract

Immunometabolism, the study of how metabolic processes influence immune cell function, has emerged as a critical field in understanding the regulation of immune tolerance and the pathological mechanisms underlying autoimmune diseases. Intracellular metabolic pathways not only provide the necessary energy for immune cell survival and activity but also shape the differentiation, phenotype, proliferation, and effector functions of immune cells. This is particularly evident in CD4+ Foxp3+ regulatory T cells (Treg), which are pivotal for maintaining immune homeostasis and preventing autoimmune reactions. Strong experimental evidence highlights the profound impact of metabolism on Treg. Their anti-inflammatory function and ability to suppress excessive immune responses depend on the integration of metabolic cues with their transcriptional and signaling networks. Iron metabolism and mitochondrial dynamics are among the key factors influencing Treg function. This review focuses on how iron and mitochondrial metabolism shape Treg biology and function.

## Introduction

Among the various T cell lineages with suppressive or regulatory functions, the CD4+Foxp3+ regulatory T cell (Treg) subset has been the primary focus of research. Treg are essential for maintaining tolerance and immune homeostasis, with altered Treg function leading to autoimmune disease and immunopathology.

This pivotal role is underscored by the association of *FOXP3* loss of function mutations with severe lymphoproliferative autoimmune disorders, such as IPEX syndrome in humans (1) and the scurfy phenotype in mice (2). These findings, combined with the identification of Foxp3 as the master regulator of the CD4+CD25+ suppressive population (3–6), have established a consistent Treg phenotype and provided essential tools for investigating this critical T cell subset. Recent studies have uncovered Foxp3-independent mechanisms contributing to Treg dysfunction, further emphasizing the complexity of their regulatory pathways (7, 8). This highlights the need to explore how various factors, particularly immunometabolism, influence Treg function. In this context, the roles of iron metabolism and mitochondrial dynamics have gained significant attention as key modulators of Treg activity.

This review focuses on how immunometabolism, with a particular emphasis on iron and mitochondrial metabolism, affects Treg function in the contexts of autoimmune diseases, hypertension-associated conditions and instead of an aging. Understanding these interactions will provide deeper insights into Treg biology and their therapeutic potential in disease management.

## Treg biology and function

Most Treg cells develop in the thymus and are classified as thymus-derived Treg (tTreg) cells (9) characterized by a T cell receptor (TCR) with relatively high affinity for self-antigen (10). Additionally, Treg cells can differentiate from conventional T cells in the periphery, forming peripherally-derived Treg cells (pTreg), or be induced *in vitro* through stimulation with IL-2 and TGF-β, known as induced Treg cells (iTreg) (11, 12), that are more prone to be specific for foreign antigen. The tTreg are widely regarded as more stable than pTreg (13). This stability issues from the thymic environment, which provides a protected setting with minimal external interference during their development and maturation. This allows sufficient time for complete epigenetic remodeling, resulting in stable and sustained Foxp3 expression.

By contrast, pTreg have a yet incomplete Treg-like epigenetic imprint, making these cells more susceptible to loss of Foxp3 expression in the presence of strong inflammation. The stability of the Treg lineage depends on continuous *FOXP3* transcription, which is maintained through changes in the methylation status of histones and CpG-rich regions. This process results in a Treg-specific hypomethylation pattern, known as the major Treg-specific demethylated region (TSDR), located within the second intron enhancer of the *FOXP3* gene, commonly referred to as conserved non-coding sequence 2 (CNS2) (14). Epigenetic modifications, such as DNA methylation and demethylation, play a crucial role in maintaining Treg stability and function. DNA demethylation can occur passively during DNA replication or actively through the catalytic activity of the ten-eleven translocation (TET) family of dioxygenases. These enzymes oxidize 5-methylcytosine (5mC) to 5-hydroxymethylcytosine (5hmC) and other intermediates, ultimately leading to the restoration of unmethylated cytosine at specific genomic positions (15, 16).

**Figure 1:**
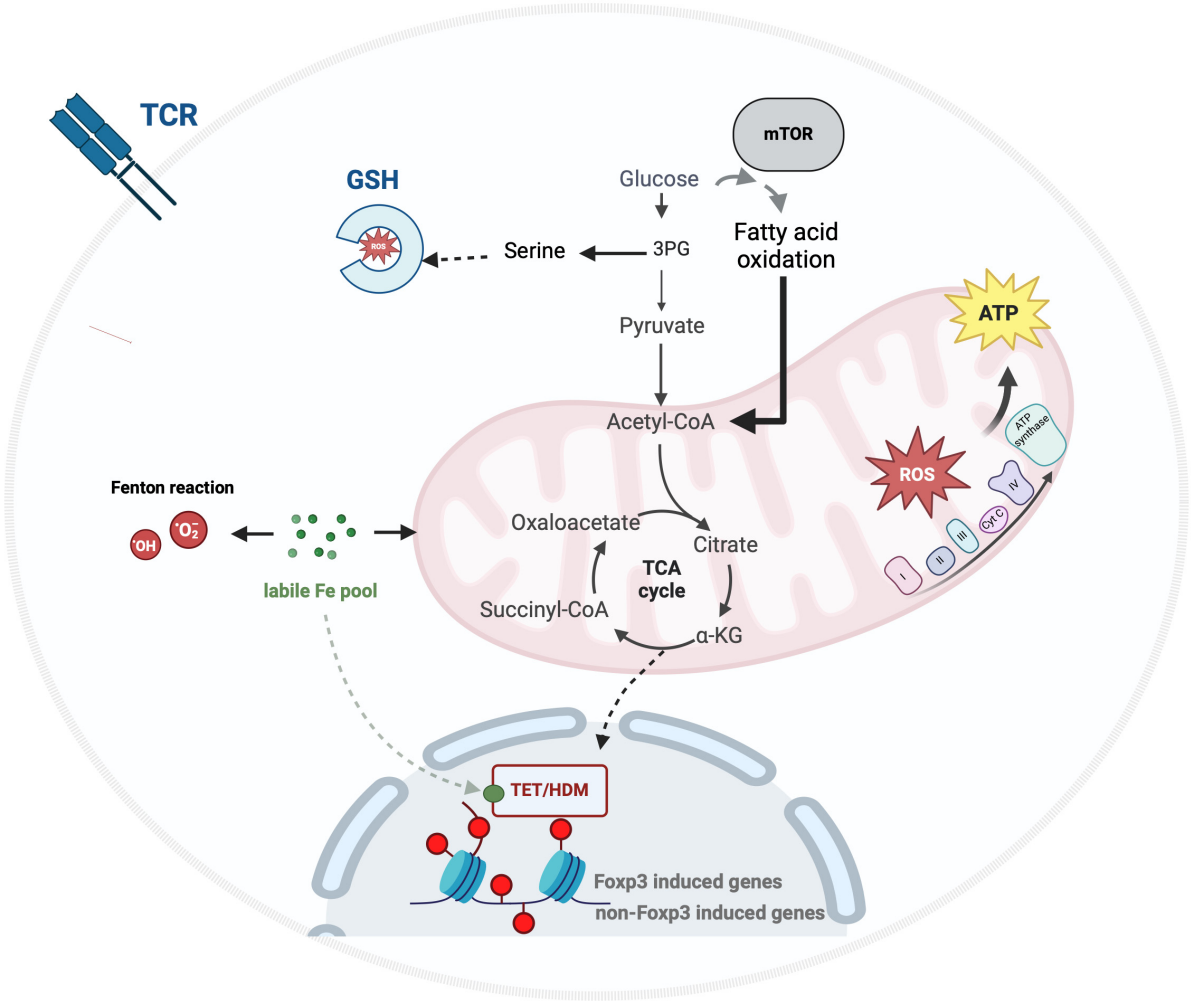
Iron and energy metabolism in Treg. Upon TCR engagement, glucose uptake by Tregs supports GSH synthesis, fuels the TCA cycle, and drives energy production through oxidative phosphorylation (OxPhos). The mammalian target of rapamycin (mTOR) pathway mediates a metabolic reprogramming of glucose metabolism towards lipid metabolism, enhancing the immunosuppressive functions of effector Tregs by facilitating metabolic and epigenetic modifications associated with iron homeostasis. TET, Ten Eleven Translocation enzyme; HDM, histone demethylase; GSH, glutathione; 3PG, 3-phosphoglycerate; ROS, Reactive Oxygen.

The demethylation mechanism is modulated by iron and key metabolites of the tricarboxylic acid cycle (TCA) that proved the profound interplay between metabolism-associated environmental signals, epigenetic modifications and Treg biology and function (17). To adapt to both intrinsic and extrinsic cues, Tregs activate a nutrient-sensing mechanism, orchestrating metabolic reprogramming to maintain and enhance their activity. Recent advances in immunometabolism have highlighted the critical role of mitochondrial dynamics and iron homeostasis in regulating and shaping Treg functionality (**Figure 1** for illustrating interplay).

## Iron metabolism in Treg

Trace element iron plays a vital role in T cell biology, serving as an essential cofactor for numerous cellular and metabolic processes. Ferroportin (FPN) and divalent metal transporter 1 (DMT1) are central to iron uptake and transfer in the duodenum, regulated adaptively by hepcidin to maintain iron homeostasis (18, 19). Since *DMT1* and *FPN* gene expression levels are highly dependent on HIF-2α, this transcription factor has been considered as a key local regulator of iron absorption, directly transactivating iron transporter genes (20).

Upon activation, T cells markedly upregulate transferrin receptor (TfR, CD71), a key protein that facilitates the uptake of transferrin-bound iron from the serum (21). This imported iron is indispensable for supporting T cell activation, proliferation, and differentiation (22, 23). Thus, iron could modulate T cell function through multiple mechanisms, including IL-2 secretion and endosomal recycling. In a model of systemic lupus erythematosus (SLE), these mechanisms may lead to iron accumulation in T cells despite SLE patients’ predisposition to low serum iron levels (23). Elevated intracellular iron, in turn, alters mitochondrial respiration and mitochondrial reactive oxygen species (ROS) production.

A recent study has shown that both human and mouse Treg cells express iron-regulatory genes, including ferritin heavy chain (*FTH*) (24). Since FTH regulates the intracellular pool of redox-active Fe²^+^, it may influence TET enzymatic activity either directly - by sequestering catalytic Fe²^+^ - or indirectly by modulating redox homeostasis. A functional link between FTH ferroxidase activity and TET function connects FTH to *FOXP3* transcription and expression, thereby impacting Treg function (25).

In addition to its role in post-transcriptional regulation, iron binds to enzymes involved in gene expression, such as TET dioxygenases, and directly interacts with certain transcription factors (26). FTH may also regulate additional local iron-dependent pathways that support Treg function, contributing to a spatial mechanism of tolerance in intestinal tissue (27–29). Thus, FTH is able to enhance HIF-2 expression, which induces c-Maf expression in Treg cells - a key factor in maintaining immunological tolerance to the microbiota. Notably, pentanoate, a microbiota-derived metabolite, may promote intestinal Treg homeostasis by modulating intracellular iron levels. Thus, in the intestine, perinatal Treg expansion is jointly linked to iron homeostasis and bacterial colonization. In a conditional CD71 knockout model, mice develop a scurfy-like disease with a complete absence of Foxp3, along with reduced bacterial proliferation due to limited iron availability (30).

The reliance of TET enzymes activity on iron availability suggests that Treg lineage stability is closely linked to cellular iron homeostasis, potentially involving a spatial mechanism of tolerance in hypermetabolic regions (31).

## Mitochondrial metabolism in Treg

Iron metabolism and iron-regulatory genes are intricately linked to TCA and oxidative phosphorylation (OxPhos) metabolism. Iron sulfur clusters acts in the catalytic centers of number of important enzymes as cytochrome oxidase, citrate synthase, aconitase and succinate deshydrogenase. Moreover, intracellular ferrous iron is a critical component of redox centers of the respiratory chain complexes driving reactive oxygen species production. Signals through TCR and IL-2 provide inputs for mammalian target of rapamycin (mTOR) activation which in turn programs the suppressive function of Treg cells and induce a metabolic shift. mTORC1 does not directly affect the expression of Foxp3 but maintain Treg functions mainly by inhibition of the mTORC2 pathway (32). Using mTOR antagonist as Rapamycin, Treg function and fatty acid oxidation were enhanced promoting both tTreg and pTreg generation through AMPK pathway (33). The reprogramming of glucose metabolism mediated by the raptor/mTORC1 pathway to lipid metabolism and OxPhos mediates the enhancement of immunosuppressive functions in effector Treg. Notably, complex III of the respiratory chain, which include the Rieske iron–sulfur subunit, is the primary site for ROS generation in both matrix mitochondria and cytosol. While ROS generation is essential for cellular signaling, decrease in ATP production and *Gpx* expression associated to excessive ROS can trigger ferroptosis, a regulated form of cell death implicated in various diseases (19). In Treg, lipid peroxidation and ferroptosis could be prevented by inducing the glutathione peroxidase Gpx4 that in turn is able to control CD71 expression and mitochondrial homeostasis (34). Thus, Gpx4 could protect activated-Treg by restoring cellular redox homeostasis.

A recent study has highlighted the metabolic-epigenetic role of respiratory chain complex III (35). Loss of complex III has been shown to result in global DNA hypermethylation without affecting the methylation status of canonical Treg genes. This effect is likely due to the accumulation of TCA metabolites such as succinate or fumarate, which inhibit TET- DNA demethylase activity by competing with the α-ketoglutarate cofactor. This inhibition disrupts Treg suppressive capacity, even without impairing their proliferation or survival. Therefore, Treg cell play a crucial role in maintaining peripheral T cell tolerance.

We are going to challenge the close relationship between iron homeostasis and energy metabolism in frequent chronic diseases and aging, in the context of Treg dysfunction.

## Autoimmune diseases

In healthy individuals, Treg exhibit enhanced mitochondrial metabolism, with the mitochondrial respiratory chain playing a pivotal role in maintaining their suppressive capacity, stability, and survival (36, 37). Conversely, in autoimmune diseases such as multiple sclerosis (MS), inflammatory bowel disease, systemic lupus erythematosus and rheumatoid arthritis (RA), Treg exhibit a significant metabolic reprogramming signature, marked by distinct features of mitochondrial dysfunction and oxidative stress-induced cell death (38). Similarly, in the experimental autoimmune encephalitis (EAE) mouse model, an MS-mouse model, Treg show increased mitochondrial oxidative stress and lysosomal dysfunction, triggering a cell death program (38). These findings suggest that mitochondrial-regulated Treg may play a key role in the onset and progression of autoimmune diseases affecting the central nervous system.

Recent advances in understanding the pathogenesis of RA highlight the intricate interplay among immune tolerance, cellular metabolism, and aging. The loss of self-tolerance, which begins decades before joint inflammation manifests, originates outside the joint. RA patients display features of premature immune aging, including mitochondrial stress, reduced ATP production, and inflammasome activation, ultimately resulting in T cell dysfunction and pyroptotic cell death (39–42). A key effector molecule in RA pathogenesis is tumor necrosis factor (TNF), which is abundantly produced in rheumatoid tissue lesions and is a well-established therapeutic target. In RA T cells, metabolic remodeling enables short-lived effector functions but compromises energy production and mitochondrial fitness, further amplifying TNF production (43). These insights place cellular metabolism and mitochondrial dynamics at the heart of RA pathogenesis, reshaping disease paradigms.

In SLE, iron metabolism plays a critical factor influencing disease progression. Excessive iron accumulation, particularly in the kidneys, plays a well-established role in exacerbating lupus nephritis (LN), a severe manifestation of SLE. This iron deposition leads to increased oxidative stress, contributing to heightened disease activity. Beyond its direct effects on the kidneys, iron may also impact immune regulation by influencing the function of Treg, which are crucial for maintaining immune tolerance. Inflammatory Treg in autoimmune diseases often exhibit heightened glycolytic metabolism, leading to impaired suppressive function (44). This dysfunction is further aggravated by iron overload, which exacerbates oxidative stress and diminishes Treg capacity (45, 46).

Promisingly, interventions targeting iron accumulation, such as iron chelators and hepcidin modulation, have shown significant improvements in LN outcomes in animal models (47). Additionally, dietary and metabolic approaches that reduce iron availability, such as low-iron diets, promote Treg expansion and survival by limiting the formation of ROS (48). By enhancing Treg function, iron chelation not only mitigates renal damage but may also help restore immune balance, thereby addressing a key mechanism of disease progression.

These findings underscore the potential of targeting metabolic pathways as a multifaceted therapeutic strategy for SLE (49). By reducing oxidative damage and enhancing Treg function, interventions aimed at regulating iron metabolism could provide innovative approaches to manage lupus nephritis and the broader systemic manifestations of SLE. Such strategies also illustrate the broader relevance of metabolic interventions in addressing the immunopathology of autoimmune diseases.

## Treg metabolism in hypertension and cardiovascular disease

Hypertension and cardiovascular diseases are profoundly influenced by the intricate interplay of immune regulation, ROS production, and metabolic reprogramming (50). Hypertension pathogenesis is influenced by multiple molecular and cellular factors, including ROS, which play a central role in the cellular processes that drive blood pressure elevation and vascular dysfunction (51). A key player in hypertension development is the renin-angiotensin system (RAS), which stimulates pathological cardiovascular remodeling (52). Overactivation of RAS contributes to endothelial dysfunction, cardiac hypertrophy, heart failure, and hypertension. The well-established link between high sodium intake and an increased risk of hypertension and cardiovascular disease further implicates immune cell metabolism in disease progression. High salt exposure affects Treg immunometabolism, impairing their suppressive function by reprogramming them into a pro-inflammatory profile through the downregulation of the OxPhos pathway (53). This metabolic reprogramming exacerbates the inflammatory response and worsens disease progression. NADPH oxidases (NOXs), particularly NOX2, are critical mediators in this process (54). These enzymes, located in cellular membranes, transport electrons to produce cytosolic ROS. While mitochondrial ROS are often implicated in oxidative stress, NOX-generated ROS are essential for modulating immune cell activities, including those of T cells. NOX2, the predominant isoform in T cells, has a dual role in regulating both effector T cells and Treg (55, 56).

Studies in mice have shown that global NOX2 deficiency leads to an increase in the number of tissue-resident Treg in the heart under normal conditions. Furthermore, NOX2 deficiency suppresses the infiltration of effector T cells, such as Th17 cells, in response to angiotensin II (Ang II), a key mediator of hypertension, resulting in reduced Ang II–induced hypertension and associated cardiac remodeling (57). These findings highlight the intricate role of NOX2 in balancing immune responses in hypertension. On one hand, NOX2 is necessary for the proper functioning of effector T cells, which contribute to inflammatory processes in hypertensive states. On the other hand, NOX2-derived ROS can impair Treg suppressive functions, undermining their protective role in mitigating inflammation and tissue damage.

Insufficient recruitment or downregulation of Treg can lead to worsened ventricular remodeling due to unchecked pro-inflammatory responses. Treg mediate immune-inflammatory regulation through mechanisms such as the release of inhibitory cytokines, IL-2 consumption, ATP/ADP depletion, and receptor-ligand interactions. In heart failure, ATP/ADP depletion, often linked to iron deficiency, induces cardiac oxidative stress, impairing the activity of antigen-presenting cells and CD8+ effector T cells (58). Promisingly, the systemic administration of exogenous Treg following myocardial infarction (MI) has been shown to enhance cardiac function in mice by reducing cardiomyocyte death and fibrosis (59). Exogenous Treg achieve this by decreasing pro-inflammatory Ly6ChiCCR2+ monocytes/macrophages and promoting a shift in macrophages toward a pro-repair phenotype.

Both adoptive Treg transfer and the expansion of endogenous Treg effectively inhibit the progression of various cardiovascular diseases, highlighting their therapeutic potential. While Treg are generally considered beneficial immune cells in transplantation and tissue repair, their role is context-dependent. Transplantation of NOX-2-deficient Treg has proved to enhances their suppressive ability and migration to heart allografts in mice (60). Amphiregulin (Areg), a Treg-derived epidermal growth factor (EGF), is critical for injury resolution (61). However, a recent study reveals that Areg also contributes to chronic rejection after organ transplantation, promoting graft vascular remodeling and fibrosis, processes exacerbated by Treg activity, oxidative stress and iron dysregulation (62).

Understanding the dual roles of Treg, particularly in inflammatory and reparative settings, is essential for developing cell therapies.

## Aging

Immunosenescence, the gradual decline in immune function with age, significantly affects Treg, leaving elderly individuals more vulnerable to infections due to suboptimal immune responses to pathogens (63). A hallmark of aging in Treg is the accumulation of reactive oxygen species, which impairs their suppressive capacity and contributes to immune dysregulation. One key molecular mechanism underlying this dysfunction involves the decline in DDB1- and CUL4-associated factor 1 (DCAF1), a crucial factor that diminishes with aging in various tissues. DCAF1 plays an essential role in maintaining Treg functionality by regulating intracellular detoxification processes. Specifically, DCAF1 interacts with glutathione-S-transferase P (GSTP1) to catalyze reactions that buffer ROS, thereby protecting Treg from oxidative damage. The disruption of the DCAF1/GSTP1/ROS axis during aging exacerbates Treg senescence, leading to impaired immune regulation, heightened inflammation, and the progression of immunological aging (64).

Inhibiting ROS accumulation represents a promising strategy to restore Treg function in the elderly. Emerging evidence suggests that iron metabolism also plays a significant role in Treg dysfunction within age. Iron promotes ROS formation through Fenton reactions, and excessive iron accumulation can exacerbate oxidative stress and lipid peroxidation in Treg. This mechanism of ferroptosis could be targeted to prevent age-related disease. In tumor cell model, GSTP1-related ferroptosis has been described with possibly similar regulatory mechanisms as for GPX4 on the ferroptosis pathway (65).

This highlights the interplay between iron homeostasis, cell senescence and immune aging. It suggests that strategies targeting iron metabolism, such as iron chelation or dietary modifications could help mitigate Treg dysfunction by reducing ROS levels. By addressing both ROS accumulation and iron dysregulation, therapeutic interventions could enhance Treg function, reduce age-associated inflammation, and improve immune resilience in the elderly. These approaches may offer new avenues for mitigating the effects of immunosenescence and promoting healthier aging.

## Conclusion

The intricate interplay between immunomodulation, Treg, iron metabolism, and energy dynamics constitutes a sophisticated regulatory network that influences hypertension, cardiovascular disease, aging and chronic rejection. Understanding these connections provides valuable insights into novel therapeutic strategies for autoimmune diseases and chronic inflammatory conditions. Future research should focus on elucidating the precise molecular mechanisms involved and translating these findings into clinical applications to optimize therapeutic outcomes.
